# Meteorological Register for October, 1817

**Published:** 1818-01

**Authors:** John Bailey

**Affiliations:** Surgeon, &c.


### METEOROLOGICAL REGISTER FOR OCTOBER, 1817,

*Kept at Harwich, in Essex,*

By JOHN BAILEY, Surgeon, &c.

| Moon's Age. | Atmospherical Preſſure. Morn. | Ev. | Temp. | Wind. | Remarks. | General Statement of Diſeaſes. |
|---|---|---|---|---|---|---|
| 1 | 29 7 | 29 7 | 55 | NE | Rain | |
| 2 | 9 | 30 | 47 | NE | | Typhus mit. |
| ☽ 3 | 30 | ½ | 53 | E | | |
| 4 | 1 | 1 | 53 | E | | Tonsilitis |
| 5 | 1½ | 1½ | 49 | E | Rain | |
| 6 | ½ | | 53 | E | Gale of Wind | Rubeola |
| 7 | | | 54 | NE | | |
| 8 | | | 52 | NE | | |
| 9 | | | 50 | SE | | |
| ● 10 | 29 8½ | 8½ | 56 | NE | Gale of Wind | |
| 11 | | | 55 | N | R. Gale of Wind | |
| 12 | | 9½ | 52 | N | ditto ditto | |
| 13 | 30 1 | 30 1½ | 49 | NE | ditto ditto | |
| 14 | 1½ | ½ | 52 | NW | Rain | |
| 15 | | 29 8½ | 50 | W | ditto | |
| 16 | 29 8 | 9½ | 49 | E | R. Gale of Wind | |
| ☽ 17 | 30 ½ | 30 | 49 | E | ditto ditto | |
| 18 | 29 8½ | 29 8½ | 47 | E | | |
| 19 | | | 47 | E | Rain | |
| 20 | | | 49 | N | Rain. Calm | |
| 21 | 8 | 8 | 53 | S | Fine and clear | |
| 22 | | | 52 | E | Cloudy | |
| 23 | ½ | ½ | 49 | E | | |
| 24 | | | 46 | NE | Rain | |
| ○ 25 | 8 | 8 | 48 | | | |
| 26 | 7 | 7 | 51 | SW | | |
| 27 | | 3½ | 51 | W | Rain | |
| 28 | 4½ | 4½ | 50 | SW | ditto | |
| 29 | | 5½ | 50 | W | | |
| 30 | 2½ | 4 | 54 | SW | Rain | |
| 31 | 5 | 8 | 50 | SW | ditto | |

Fevers of the typhoid kind have prevailed pretty much among the lower classes of the community in this town and neighbourhood. Many young children have been affected by it, and several have died. In adults, it has run on for many days without appearing to receive any check from the usual means of relief.

Saline purgatives, combined with repeated nauseating doses of antimonial powder, in the form of bolus, washed down with an oz. of Minderus's spirit; blisters to the nape of the neck; pediluvia; frequent washing of the hands, face and neck; with occasional pills of calomel in doses of five or six grains, *hora somni*, when the bowels have not answered during the day to other aperients, have been the means which your Reporter has commonly, and for the most part successfully, resorted to.

Tonsilitis of a contagious kind, unaccompanied by exanthemata, has likewise prevailed.

